# Physical and psychosocial working conditions as predictors of 5-year changes in work ability among 2078 employees in Germany

**DOI:** 10.1007/s00420-021-01716-9

**Published:** 2021-06-27

**Authors:** Hermann Burr, Stefanie Lange, Marion Freyer, Maren Formazin, Uwe Rose, Martin Lindhardt Nielsen, Paul Maurice Conway

**Affiliations:** 1 Federal Institute for Occupational Safety and Health (BAuA), Nöldnerstraße 40-42, 10317 Berlin, Germany; 2 Lægekonsulenten.Dk, AS3 Companies, Hasselager Centervej 35, 8260 Viby J, Denmark; 3 University of Copenhagen, Øster Farimagsgade 2A, 1353 Copenhagen, Denmark

**Keywords:** Prospective study, Psychosocial risk factors, Physical demands, COPSOQ

## Abstract

**Objective:**

To examine 5-year prospective associations between working conditions and work ability among employees in Germany.

**Methods:**

A cohort study (2011/2012–2017), based on a random sample of employees in employments subject to payment of social contributions aged 31–60 years (Study on Mental Health at Work; S-MGA; *N* = 2,078), included data on physical and quantitative demands, control (influence, possibilities for development, control over working time), relations (role clarity and leadership quality) and work ability (Work Ability Index, WAI; subscale ‘subjective work ability and resources’). Data were analysed using linear regression.

**Results:**

Physical demands and control were associated with small 5-year changes in work ability (Δ*R*^2^ = 1%). Among the subgroup of employees with ≥ 25 sickness days, possibilities for development, control and quality of leadership were associated with changes in work ability (Δ*R*^2^ = 8%).

**Conclusions:**

The impact of working conditions on long term changes in work ability seems to be negligible. However, in vulnerable subpopulations experiencing poor health, working conditions may be associated to a larger extent to work ability over this time span.

## Introduction

Promoting and maintaining work ability is a main goal for employees and employers, as well as for policy makers and social security systems. The effective management of work ability has a primary role in reducing disability costs and securing gainful employment for the workers, productive workplaces for the employers, and a healthy economy for society. To reach this goal, in-depth knowledge is needed of factors influencing work ability, including working conditions.

A widely used measure of work ability is the Work Ability Index (WAI) (van den Berg et al. 2009). This measure is based on the definition of work ability as an individual’s current and future potential to handle his/her work tasks given his/her pool of physical and psychological resources (Ilmarinen et al. 2008). Work ability has multiple determinants, including health and functional capacities, competence, values, attitude and motivation, and working conditions (Ilmarinen et al. 2005). The present study focuses on physical and psychosocial working conditions as possible risk factors for reduced work ability. Being more easily modifiable than individual factors, work-related antecedents of work ability play a major role in the promotion and prevention of work ability.

There is a large body of cross-sectional research examining the relationship between working conditions and work ability, which was summarized in two literature reviews (Cadiz et al. 2018; van den Berg et al. 2009). These have identified a range of both physical (e.g., demanding work postures, heavy lifting) and psychosocial (e.g., quantitative and emotional demands, influence at work, possibilities for development) factors in association with work ability. However, cross-sectional studies suffer from a limited internal validity, given that relationships between working conditions and work ability are bidirectional (Cadiz et al. 2018). Longitudinal designs are, therefore, needed to determine the directionality of causal relationships (Taris and Kompier 2014; Zapf et al. 1996).

We performed a literature review of the existing studies that examined baseline working conditions as risk factors for changes in work ability at follow-up (Airila et al. 2014; Bethge and Radoschewski 2012; Bethge et al. 2012; Boschman et al. 2017; Boström et al. 2012; Camerino et al. 2008; Emberland and Knardahl 2015; Feldt et al. 2009; Leijon et al. 2017; Martinez et al. 2016; McGonagle et al. 2015; Oakman et al. 2019; Punakallio et al. 2019; Rongen et al. 2014; Spanier et al. 2018; Sugimura and Thériault 2010; Tonnon et al. 2019; Tuomi et al. 2001, 1997, 2004; Weber et al. 2020). In all, we identified 21 studies. We excluded three of these (Boström et al. 2012; Tuomi et al. 1997, 2004) as they examined associations between changes in working conditions and changes in work ability, making causal conclusions difficult to establish. The remaining 18 longitudinal studies investigated the associations between baseline working conditions and changes in work ability from baseline to follow-up. The results of these studies are summarised in Table 1.

**Table 1:**
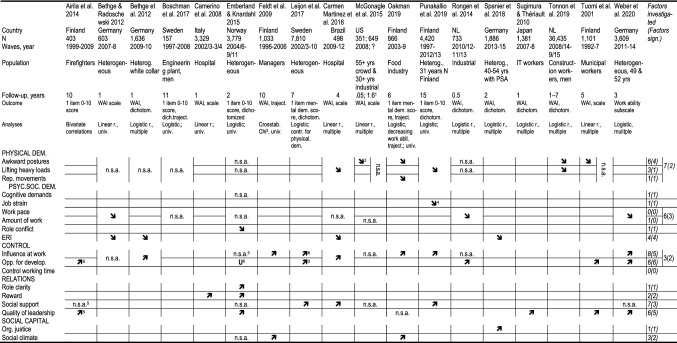
Work environment risk factors for work ability considered in 18 longitudinal studies

*PSA* Previous sickness absence, *n.s.a* No significant association

^1^Crowd worker cohort 2–3 weeks; industrial worker cohort 1.6 years

^2^Only for 30 + aged industrial workers, not significant association for 55 + aged crowd workers

^3^The two QPS scales control over work intensity and decision control – neither predicted work ability

^4^Only women – regarding men no significant association

^5^Based on crude correlations

^6^The QPS-Nordic scale Positive challenge (mix of opportunities for development and meaning of work). U-shaped correlation

^7^Only men;—regarding women no significant association

^8^Only among those without physical strenuous work. Results on job strain show that it all is due to low influence at work

Of the reviewed studies, three were based on heterogeneous populations covering employees aged 18–30 years to 55–64 years. Another three studies relied on heterogeneous populations but examined specific birth cohorts; of these, one study focused on employees with prior long-term sickness absence. The remaining 12 studies were based on specific occupational sectors or industries. All but one study (Tonnon et al. 2019) included psychosocial working conditions as antecedents of work ability, while 11 considered also physical working conditions. Most studies focused on psychosocial factors such as quantitative demands, influence at work, possibilities for development, social support and quality of leadership, but paid little attention to other factors, including lifting heavy loads, repetitive movements, control over working time, role conflicts, role clarity, rewards and organizational justice. However, most of the few studies that have examined these factors found these to be associated with changes in work ability. Other studies combined factors into aggregated measures, making it difficult to disentangle the effects of individual risk factors. In particular, the aggregation of physical demands into a global measure resulted in a limited scrutiny of the specific impact of factors such as lifting heavy loads and repetitive movements. Similarly, aggregating quantitative demands into a global measure did not allow to estimate the effects of specific facets of demand such as work pace and amount of work. All the physical and psychosocial factors mentioned above have been associated with health outcomes, including musculoskeletal disorders (da Costa and Vieira 2010) and depressive symptoms (Theorell et al. 2015), respectively. In turn, both health outcomes have been found in association with reduced work ability (Koskinen et al. 2008).

Another common thread of the reviewed studies is that duration of exposure is rarely considered when examining the effects of working conditions on health-related outcomes (Taris and Kompier 2014), including work ability. Yet, it can be expected that the risk of impaired work ability increases when the duration of exposure to adverse working conditions is longer. Supporting this, previous studies found evidence that a longer duration of exposure to job strain (an indicator or unfavourable working conditions) is associated with a higher risk of depressive symptoms and coronary heart disease (Kivimäki et al. 2006; Madsen et al. 2017). In the few longitudinal studies that considered the effect of duration of exposure, such effect was estimated by means of either retrospective, self-report measures of change in exposure between baseline and follow-up (Tuomi et al. 2001) or using analyses of simultaneous changes in risk factors and outcome (Boström et al. 2012; Tuomi et al. 1997, 2004). Given these limitations, more studies are needed to shed light onto the role of duration of exposure to working conditions in relation to changes in work ability.

Finally, it can be expected that the effects of working conditions on work ability depend on a worker’s health status. Previously, only one longitudinal studies has examined if health interacts with working conditions in predicting work ability (Neupane et al. 2013). Also, earlier studies have shown that working conditions play a stronger role in relation to early labour market exit among employees with a poorer health (Boot et al. 2014; de Boer et al. 2018; Jonsson et al. 2019). This is also supported by studies examining the effects of working conditions on chronic diseases; when exposed to poor working conditions, workers with a disease are at a higher risk of developing new diseases than their disease-free counterparts (Kivimaki et al. 2018; Kivimaki and Steptoe 2018).

In this 5-year prospective study in Germany, we therefore aimed to examine the effects on work ability of a range of psychosocial and physical working conditions—including a number of under investigated factors, namely lifting heavy loads, repetitive movements, work pace, amount of work and role clarity. In addition, we investigated whether these effects were stronger with a longer duration of exposure and whether they were dependent on health status.

## Methods

### Population

**Fig. 1:**
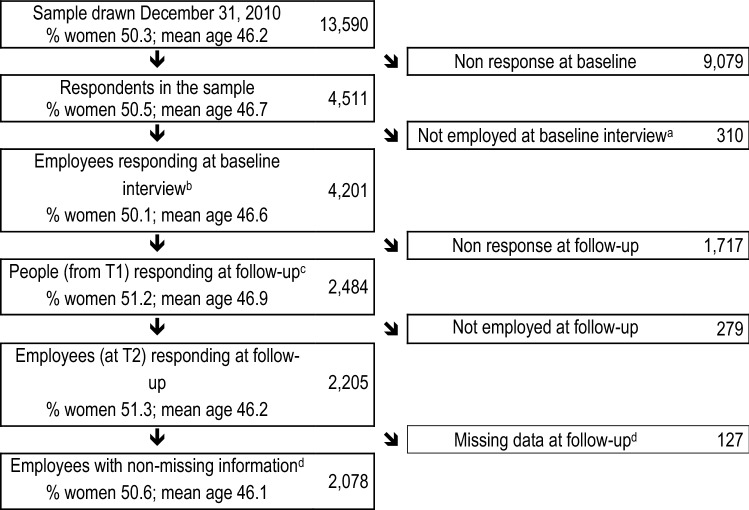
Flow diagram of participation. ^a^13 months (range 11–17) passed between sampling date and baseline interviews date; in this period, 310 people ceased to be employees. ^b^Mean baseline interview date January 2012. ^c^Mean follow-up interview date August 2017. ^d^Regarding the following variables: gender, age, SES, working conditions, work ability and sickness days

We used data from the Study on Mental Health at Work (S-MGA), a German nation-wide panel study (baseline: 2011/2012, follow-up: 2017) (Rose et al. 2017). At baseline, the target population was represented by all currently employed individuals aged 31–60 years in Germany (currently employed are defined as those working citizens subject to mandatory social contributions. Workers in the target population constituted 80% of all economically active citizens of Germany in this age range in 2012 (Statistisches Bundesamt (destatis) 2021a; Statistisches Bundesamt (destatis) 2021b). The remaining 20% not included in the target population consisted of the self-employed and civil servants. The advantage of using this sample frame was that it enabled attrition analyses. The study sample was drawn from the target population in the Integrated Employment Biographies register on the reference date of December 31 2010 (Rose et al. 2017). Overall, 13,590 people were randomly selected and then contacted. Of the 4,511 respondents who took part in the computer-assisted personal interviews at baseline (response: 33%), 4,201 were employed. Among these, 2,484 took also part in the follow-up interviews (Fig. 1). Of these, 2,205 were still employed at follow-up. We further excluded those respondents with missing values on gender, age, SES, working conditions, work ability and sickness days, leading to a final cohort sample of 2,078 respondents, which constituted the cohort sample included in the present study (follow-up response: 53%, estimated cohort response 19%, see Table 2). Response in the cohort sample was independent of gender, but lower among the younger and unskilled workers than among the older and professionals/managers (Table 2). Response at follow-up was only marginally associated with baseline level of work ability (Chi^2^-test; p for the whole variable = 0.075; 1st (lowest) quartile of work ability 57%, 2nd quartile: 60%, 3rd quartile = 59%, 4th (highest) quartile = 63%; not shown). There were no notable differences in relation to physical and psychosocial working conditions between the baseline and the cohort samples (Table 3).

**Table 2:** Response in interviews at baseline, at follow-up and in the cohort by gender, age and SES

	Baseline response ^a^; %		Follow-up response among baseline employees ^b^, %		Estimated cohort response fraction of the drawn sample ^c^, %	
	*p* value^d^	%	*p* value ^d^	%	*p* value ^d^	%
Gender	0.746		0.999		0.151	
Men		33		53		17
Women		33		53		18
Age	**0.000**		0.250		**0.000**	
55–60		**39**		49		**19**
49–54		**35**		54		**19**
43–48		**33**		53		**17**
37–42		**32**		55		**17**
31–36		**27**		52		**14**
SES	**0.000**		**0.000**		**0.000**	
Academics, managers		**38**		**60**		**23**
Semi-professionals		**38**		**65**		**25**
Skilled workers		**32**		**59**		**19**
Unskilled workers		**29**		**51**		**15**
Total		33		53		19

Bold indicates significant *p*-values and response %

Siginificance level *p* = 0.05 (Rothman 1990).The table is based on published baseline and follow-up attrition analyses (Rose et al. 2017; Schiel et al. 2018) and response fractions in the analysed cohort of the present paper, see also Fig. 1

^a^Fraction responded at baseline (*n* = 4511) of the drawn sample (*n* = 13,590)

^b^Fraction responded at follow-up and with non-missing information (*n* = 2078) of the employees who responded at baseline who still were employees at follow-up (*n* = 3922), that is censoring employees at baseline who at follow-up ceased to be employees (*n* = 279)

^c^Fraction in the analysed cohort (2078) of the drawn sample (estimated by multiplying the fraction responding at baseline with the fraction responding at of follow-up)

^d^This *p* value denotes to what extent the whole categorical variable is associated with response (Chi^2^ test)

**Table 3:** Characteristics of the sample of employees at baseline and of the analysed cohort

	Employees responding at baseline		Analysed cohort^a^	
	*N (%)*	*M* (*SD*)	*N (%)*	*M* (*SD*)
Gender				
Men	2096 (50)		1026 (49)	
Women	2105 (50)		1052 (51)	
Age		46.6 (7.8)		46.1 (7.3)
SES				
Unskilled workers	282 (7)		119 (6)	
Skilled workers	1892 (45)		845 (41)	
Semi-professionals	1099 (26)		594 (29)	
Academics/managers	928 (22)		520 (25)	
Physical demands (1–5)^b^				
Standing/walking		2.9 (1.5)		2.8 (1.5)
Awkward body postures		1.7 (1.1)		1.7 (1.1)
Carrying and lifting		1.8 (1.1)		1.7 (1.0)
Repetitive movements		2.4 (1.5)		2.3 (1.5)
Quantitative demands (1–5)^b^				
Work pace		3.7 (1.0)		3.7 (1.0)
Amount of work		2.8 (0.9)		2.8 (0.9)
Control (1–5)^b^				
Influence at work		2.7 (1.0)		2.7 (0.9)
Possibilities for development		3.6 (0.9)		3.7 (0.9)
Control over working time		3.2 (1.1)		3.3 (1.0)
Relations (1–5)^b^				
Role clarity		4.3 (0.6)		4.3 (0.6)
Quality of leadership		3.3 (0.9)		3.3 (0.9)
Work ability at baseline (4–31)^b^		25.9 (4.2)		26.4 (3.9)
Total	4201		2078	

^a^Employed at baseline and follow-up and with non-missing information on gender, age, SES, working conditions, work ability and sickness days (Fig. 1)

^b^Numbers in parentheses show the possible range of items or scales

### Measures

All information was obtained through computer-assisted personal interviews at the respondents’ home (Rose et al. 2017).

### Dependent variable

*Work ability* at baseline and follow-up: We calculated a sum score ranging from 4 to 31 based on four items taken from the WAI, covering subjective work ability and resources (Ilmarinen 2009; McGonagle et al. 2015; Tuomi et al. 2003). The four items were: ‘Current work ability compared with the lifetime best’ (WAI1), ‘Work ability in relation to the demands of the job’ (WAI2), ‘Own prognosis of work ability 2 years from now’ (WAI6) and ‘Mental resources’ (WAI7) (Freyer et al. 2019). In contrast to common WAI procedures for score calculation, each of the four items contributed equally to the scale. The Cronbach’s alpha of the scale at baseline was 0.73 and the inter-item correlations ranged from 0.31 to 0.59–at follow-up alpha was 0.74 and inter-item correlations ranged from 0.28 to 0.60. Means and standard deviations can be seen in Table 3.

We decided not to include the WAI items measuring health. Several studies demonstrated a two-factor structure for the WAI, with one factor indicating work ability proper and the other indicating health (e.g., Alexopoulos et al. 2013; Freyer et al. 2019; Martus et al. 2010; Radkiewicz and Widerszal-Bazyl 2005). In particular, in previous studies a factor including the four WAI items not considering health-related dimensions, revealed a better predictive validity on relevant outcomes such as work disability (Alavinia et al. 2009; Ilmarinen and Tuomi 2004).

### Independent variables

The working conditions considered in the present study include the following four domains: physical demands, quantitative demands, job control and relations at work. All scales and single item measures ranged from 1 to 5. Scale scores were calculated if at least half of their items were answered (Nübling et al. 2006; Pejtersen et al. 2010). Means and standard deviations of all scales are shown in Table 3.

### Physical demands

This domain included standing posture, sitting posture, awkward body postures, carrying and lifting and repetitive movements. These were measured by five items taken from the BiBB/BAuA employment study (Hall et al. 2007; Tynes et al. 2017): “How often do you have to …—work in a standing position?”, “- work in a sitting position?”, “- work in a bent, squatted, kneeling, lying or overhead position?”, “-carry or lift heavy loads (women > 10 kg, men > 20 kg)?”, “- do repetitive movements (one-sided physical work)?”. The response options were “never”, “up to 1/4 of the time”, “up to half of the time”, “up to 3/4 of the time”, “more than three quarters (almost all of the time)”. Due to the high inter-correlations between the two items measuring standing and sitting (reverse coded; *r* = 0.90), these were combined into a single scale called ‘standing/walking’, which was calculated as the mean of the two items. Cronbach’s alpha for this scale was 0.95. The other three items were considered as separate dimensions.

### Quantitative demands

This domain included the single-item measure work pace and the scale amount of work (COPSOQ; Kristensen et al. 2005; Nübling et al. 2005).

*Work pace* was assessed through the single item: “Do you have to work very fast?” (Kristensen et al. 2004). The response options were “always”, “often”, “sometimes”, “seldom”, “never / hardly ever”.

*Amount of work* was assessed with a five-item scale (Kristensen et al. 2004): “How often …—is your workload unevenly distributed so it piles up?”, “- do you not have time to complete all your work tasks?”, “- do you get behind with your work?”, “- do you have enough time to complete all your work tasks?” (reversely coded), “- do you have to do overtime?”. The response options were the same as for work pace. Cronbach’s alpha was 0.82, range of inter-item correlations: 0.32–0.69).

### Job control

This domain encompassed the three scales influence at work, possibilities for development and control over working time (COPSOQ; Kristensen et al. 2005; Nübling et al. 2005). Items of the first and the third scale had the same response options as the items used to measure work pace. The items of the second scale had the response options “to a very large extent”, “to a large extent”, “somewhat”, “to a small extent”, “to a very small extent”.

*Influence at work* was assessed with the four items: “How often …—do you have a large degree of influence on the decisions concerning your work?”, “- do you have a say in choosing who you work with?”, “- can you influence the amount of work assigned to you?”, “- do you have any influence on what you do at work?” (Cronbach’s alpha = 0.70, range of inter-item correlations: 0.31–0.43).

*Possibilities for development* were assessed with the two items: “Do you have the possibility of learning new things through your work?” and “Can you use your skills or expertise in your work?” (Cronbach’s alpha = 0.61, inter-item correlation: 0.44).

*Control over working time* was assessed with the four items: “How often …—can you decide when to take a break?”, “- can you take holidays more or less when you wish?”, “-can you leave your work to have a chat with a colleague?” and “If you have some private business is it possible for you to leave your piece of work for half an hour without special permission?” (Cronbach’s alpha = 0.71, range of inter-item correlations: 0.23–0.49).

### Relations at work

This domain encompassed the two scales role clarity and quality of leadership (COPSOQ; Kristensen et al. 2005; Nübling et al. 2005). The items in these scales had the same response options as the items used to measure possibilities for development.

*Role clarity* was assessed with the three items: “Do you know exactly how much say you have at work?”, “Does your work have clear objectives?” and “Do you know exactly which areas are your responsibility?” (Cronbach’s alpha = 0.69, range of inter-item correlations: 0.36–0.52).

*Quality of leadership* was assessed with the four items: “To what extent would you say that your immediate superior …—makes sure that the individual member of staff has good development opportunities? “, “- gives high priority to job satisfaction? “, “- is good at work planning?”, “− is good at solving conflicts?” (Cronbach’s alpha = 0.85, range of inter-item correlations: 0.53–0.66).

We decided not to include *social support* given its limited content validity, as the items of the scale version used in this study measured both experienced and needed amount of support (Burr et al. 2019).

### Sickness days

Sickness days prior to baseline were used as a measure of health status and consisted of a single item: ‘How many full days have you been actually sick in the last 12 months, regardless of whether you were on sick leave or not?’. To the best of our knowledge, this measure has not been previously validated. The answers were categorized into 0–24 days (*n* = 1833) and ≥ 25 days (*n* = 248). We chose this cut-off point as it identified those 10% of the sample having reported the highest amount of sickness days.

### Employment stability

Employment stability (i.e., whether the respondent stayed or changed employment from baseline to follow-up) was used as a proxy measure for duration of exposure to working conditions. We measured employment stability through questions on employment biographies between baseline and follow-up (introductory question: ‘In our last interview, you stated that you in your main occupation were …’ with the response options: ‘Activity shown is correct’ ‘Activity shown is not correct’) (Borsch-Supan et al. 2013). Staying in the same employment was previously shown to be associated with stability of exposure to working conditions, whereas leaving the job was associated with changes in exposure (Garthe and Hasselhorn 2020). In the present S-MGA-cohort, associations between working conditions at baseline and follow-up were in all but two cases significantly stronger among those employees who stayed in their employment than among employees who changed employment. In only two cases (carrying/lifting and role clarity) associations were the same in the two groups. This indicates that employment stability is a valid proxy for duration of exposure (Appendix Table A).

### Covariates

We included age and socioeconomic status (SES) at baseline as potential confounders. Gender was not included as it was not associated with work ability. SES was operationalized via the respondents’ occupations, which were coded manually according to the International Standard Classification of Occupations (ISCO 08) and categorized into four groups based on skill levels: unskilled workers, skilled workers, semi-professionals, academics/managers (Hagen 2015).

Table 4 shows the associations between all physical and psychosocial working conditions, gender, age, SES (treated as a linear variable), and work ability at baseline. The highest correlations were found between standing/walking, awkward body postures and carrying/lifting; the three next highest correlations were between standing/walking and SES; between work pace and amount of work; and between control over working time and standing/walking. Note also that baseline work ability was correlated with baseline working conditions, with correlations ranging from ± 0.10 to ± 0.30.

**Table 4:** Pearson inter correlations between working conditions, age and SES at baseline among 2078 employees aged 31 to 60 years in Germany

		Demographics			Physical demands				Quantitative demands		Control			Relations	
		Gender	Age	SES	Standing/walking	Awkw. body post	Carrying/lifting	Rep. movements	Work pace	Amount of work	Influence at work	Poss. for develop	Contr. work. time	Role clarity	Qual. Leadership
Demographics	Age	0.05*													
	SES	−0.02	−0.05*												
Physical demands	Standing/walking	−0.03	−0.02	−0.44**											
	Awkward body postures	−0.06**	−0.07**	−0.31**	0.54**										
	Carrying/lifting	−0.04	−0.07**	−0.31**	0.55**	0.59**									
	Repetitive movements	0.08**	0.00	−0.09**	−0.14**	0.01	0.04								
Quantitative demands	Work pace	0.10**	−0.09**	−0.05*	0.06**	0.11**	0.17**	0.15**							
	Amount of work	−0.08**	−0.10**	0.26**	−0.14**	−0.01	0.04*	0.12**	0.41**						
Control	Influence at work	−0.15**	−0.02	0.28**	−0.07**	−0.02	−0.09**	−0.18**	−0.12**	−0.03					
	Possibilities for development	−0.08**	−0.04*	0.40**	−0.17**	−0.04	−0.06*	−0.10**	−0.04	0.16**	0.38**				
	Control over working time	−0.20**	−0.05*	0.22**	−0.41**	−0.26**	−0.30**	−0.05*	−0.17**	−0.03	0.36**	0.22**			
Relations	Role clarity	0.05*	0.08*	0.00	0.05*	0.05*	−0.03	−0.03	−0.01	−0.13**	0.12**	0.23**	0.05*		
	Quality of leadership	0.06**	0.01	−0.02	0.04	0.04	−0.02	−0.08**	−0.11**	−0.26**	0.18**	0.23**	0.09**	0.22**	
Work ability		0.00	−.14**	.18**	−.21**	−.23**	−.26**	−.14**	−.12**	−.20**	.21**	.24**	.25**	.18**	.24**

*p value < 0.05; ** *p* value < 0.01

### Statistical analyses

We applied linear regression analyses to examine the association between physical and psychosocial working conditions at baseline and work ability at follow-up. We ran two sets of linear regression analyses for every physical and psychosocial working condition separately. In model 1, we adjusted only for age and SES at baseline. In model 2, we adjusted for work ability at baseline to estimate the effects of psychosocial working conditions on changes in work ability during follow-up. We did not calculate mutually adjusted regression coefficients to avoid collinearity, which can occur at inter-correlations as low as 0.25 even when as few as three independent variables are involved (Table 4) (Vatcheva et al. 2016). We performed mutually adjusted analyses only to calculate additional explained variance (Vatcheva et al. 2016).

A first set of sensitivity analyses was carried out to examine the effect of duration of exposure to adverse working conditions on work ability. Specifically, we reran the main analysis while excluding all respondents who changed their employment between baseline and follow-up (*N* = 1653).

A second set of sensitivity analyses was performed to investigate if the effects of working conditions were dependent on the amount of sickness days. First, we repeated the main analysis while stratifying by sickness days. Second, we tested the interaction between working conditions and sickness days.

For all analyses, the significance level was set to 0.05. No Bonferroni adjustments were applied (Rothman 1990).

## Results

Table 5 shows the individual associations between each of the working conditions at baseline and work ability at follow-up. In model 1, adjusted for age and SES at baseline, all physical and quantitative demands were associated with a decreased level of work ability, while all factors within the job control and relational domains were associated with increased levels of work ability. Taken together, all factors explained 9% of the variance in work ability. In model 2, additionally adjusted for work ability at baseline, the strength of associations between each of the work environment dimensions and work ability decreased considerably (working conditions were correlated with baseline work ability; Table 3. Baseline work ability predicted work ability at follow-up, with an explained variance of *R*^2^ = 0.25 (table not shown). Only standing/walking, awkward body postures, carrying/lifting and control over working time were still significantly associated with work ability at follow-up. The variance in work ability explained by all factors together decreased to 1%.

**Table 5:** Associations between baseline working conditions and work ability five years later among 2,078 employees aged 31 to 60 years in Germany. Linear regressions

Domain	Dimension	Model 1. Adjusted for age and SES at baseline		Model 2. Adjusted for age, SES and work ability at baseline	
		Δ*R*^2^ ^a^	Beta (95% CI)	Δ*R*^2^ ^a^	Beta (95% CI)
ALL	All	0.088^b^		*0.012*^b^	
Physical demands	Standing/walking	0.013	**−0.13 (−0.18;** −**0.08)**	*0.002*	−**0.06 (**−**0.10;** −**0.01)**
	Awkward body postures	0.020	−**0.15 (**−**0.19; **−**0.11)**	*0.003*	−**0.06 (**−**0.10; −0.02)**
	Carrying/lifting	0.031	**−0.19 (−0.23; −0.14)**	*0.006*	−**0.08 (−0.12; −0.04)**
	Repetitive movements	0.007	−**0.09 (**−**0.13; **−**0.04)**	*0.001*	−0.03 (−0.07; 0.01)
Quantitative demands	Work pace	0.002	−**0.04 (**−**0.09; **−**0.00)**	*0.000*	0.02 (−0.02; 0.05)
	Amount of work	0.021	−**0.15 (**−**0.19; **−**0.11)**	*0.000*	−0.02 (−0.06; 0.02)
Control	Influence at work	0.009	**0.10 (0.06; 0.14)**	*0.000*	0.02 (−0.02; 0.06)
	Possibilities for development	0.012	**0.12 (0.08; 0.17)**	*0.001*	0.03 (−0.01; 0.07)
	Control over working time	0.026	**0.17 (0.12; 0.21)**	*0.004*	**0.07 (0.03; 0.11)**
Relations	Role clarity	0.012	**0.11 (0.07; 0.15)**	*0.000*	0.02 (−0.02; 0.06)
	Quality of leadership	0.015	**0.12 (0.08; 0.16)**	*0.000*	0.01 (−0.03; 0.05)

Siginificance level *p* = 0.05 (Rothman 1990). Bold values denote significant beta regression coefficients

^a^Δ*R*^2^ indicates the change of explained variance (*R*^2^) in comparison to a model with adjustment variables only

^b^Model with all working conditions entered simultaneously

### Sensitivity analyses

#### Employment stability

When repeating the analyses in the sample of employees who remained in the same employment during follow-up (Table 6), the associations were generally stronger than those observed in the full sample. The variance explained in work ability at follow-up was 11% for the model adjusted for age and SES only (Model 1), and 2% for the model additionally adjusted for work ability at baseline (Model 2).

**Table 6:** Associations between baseline working conditions and work ability 5 years later among 1698 employees aged 31–60 years in Germany without change in employment relationship during follow-up

Domain	Dimension	Model 1. Adjusted for age and SES at baseline		Model 2. Adjusted for age, SES and work ability at baseline	
		Δ*R*^2^ ^a^	Beta (95% CI)	Δ*R*^2^ ^a^	Beta (95% CI)
ALL	All	0.108^b^		*0.020*^b^	
Physical demands	Standing/walking	0.015	−**0.13 (**−**0.19;** −**0.08)**	*0.003*	−**0.06 (**−**0.10; −0.01)**
	Awkward body postures	0.017	−**0.14 (**−**0.19;** −**0.09)**	*0.001*	−0.03 (−0.08; 0.01)
	Carrying/lifting	0.036	−**0.20 (**−**0.25;** −**0.15)**	*0.006*	−**0.08 (**−**0.13; −0.04)**
	Repetitive movements	0.006	−**0.08 (**−**0.13;** −**0.03)**	*0.000*	−0.02 (−0.06; 0.02)
Quantitative demands	Work pace	0.002	−**0.05 (−0.09;** −**0.00)**	*0.000*	0.02 (−0.02; 0.06)
	Amount of work	0.024	−**0.16 (**−**0.20;** −**0.11)**	*0.001*	−0.03 (−0.08; 0.01)
Control	Influence at work	0.009	**0.10 (0.05; 0.15)**	*0.001*	0.03 (−0.01; 0.07)
	Possibilities for development	0.020	**0.15 (0.10; 0.20)**	*0.003*	**0.06 (0.02; 0.10)**
	Control over working time	0.032	**0.18 (0.14; 0.23)**	*0.008*	**0.09 (0.05; 0.13)**
Relations	Role clarity	0.018	**0.14 (0.09; 0.18)**	*0.002*	**0.05 (0.01; 0.09)**
	Quality of leadership	0.024	**0.16 (0.11; 0.20)**	*0.002*	**0.04 (0.00; 0.08)**

Linear regressions

Siginificance level *p* = 0.05 (Rothman 1990). Bold values denote significant betas

^a^Δ*R*^2^ shows the change of explained variance (*R*^2^) in comparison to a model with adjustment variables only in the respective model

^b^For a model with all working conditions simultaneously

#### Sickness days

When repeating the main analysis in strata defined by number of sickness days (Table 7), significant interactions between the dichotomized sickness days variable and working conditions were found regarding possibilities for development (*p* = 0.003), control over working time (*p* = 0.006), role clarity (*p* = 0.027), and quality of leadership (*p* = 0.002). Among employees with < 25 sickness days, the variance explained by all working conditions together was 1%. Among these, high physical demands (standing/walking, awkward body postures and carrying/lifting) were associated with decreased work ability, whereas control over working time was associated with increased work ability. Among employees with ≥ 25 sickness days, the total variance explained by working conditions was 8%. In this stratum, possibilities for development, control over working time and quality of leadership were associated with increased work ability.

**Table 7:** Associations between baseline working conditions and work ability 5 years later stratified by sickness days prior to baseline among 2,078 employees aged 31 to 60 years in Germany. Linear regressions

Domain	Dimension	Interaction with sickness days, *p*^a^	Strata defined by sickness days in the year prior to baselineAdjusted for age, SES and work ability at baseline			
			0–24 sickness days^a^ *N* = 1.830		≥ 25 sickness days^a^ *N* = 248	
			*ΔR*^2^ ^b^	Beta (95% CI)	*Δ R*^*2*^ ^b^	Beta (95% CI)
All	All		0.011^*c*^		*0.082*^*c*^	
Physical demands	Standing/walking	0.844	0.002	**−0.06 (−0.10; −0.01)**	*0.002*	−0.05 (−0.17; 0.07)
	Demanding body post	0.443	0.003	**−0.06 (−0.10; −0.02)**	*0.001*	−0.03 (−0.14; 0.08)
	Carrying/lifting	0.918	0.005	**−0.08 (−0.12; −0.03)**	*0.009*	−0.10 (−0.22; 0.01)
	Repetitive movements	0.227	0.002	−0.04 (−0.08; 0.00)	*0.002*	0.04 (−0.07; 0.15)
Quantitative demands	Work pace	0.345	0.000	0.01 (−0.04; 0.05)	*0.004*	0.07 (−0.04; 0.18)
	Amount of work	0.977	0.000	−0.02 (−0.07; 0.02)	*0.000*	−0.02 (−0.13; 0.10)
Control	Influence at work	0.222	0.000	0.02 (−0.02; 0.06)	*0.001*	0.03 (−0.09; 0.14)
	Poss. for development	0.003	0.000	0.01 (−0.04; 0.05)	*0.018*	**0.14 (0.03; 0.26)**
	Control over working time	0.006	0.002	**0.05 (0.01; 0.09)**	*0.025*	**0.16 (0.05; 0.27)**
Relations	Role clarity	0.027	0.000	0.00 (−0.04; 0.04)	*0.011*	0.10 (−0.00; 0.21)
	Quality of leadership	0.002	0.000	−0.01 (−0.05; 0.03)	*0.014*	**0.12 (0.01; 0.23)**

Bold values indicate significant interactions of betas

Siginificance level *p* = 0.05 (Rothman 1990). Bold values denote significant p values (1st column) or betas (3rd, and 5th column)

^a^*p* for interaction with sickness days in the year prior to baseline as risk factor for work ability 5 years later

^b^Δ*R*^2^ shows the change of explained variance (*R*^2^) in comparison to a model with adjustment variables only

^*c*^For a model with all working conditions

## Discussion

A main finding of our study is that, in healthy working populations, the impact of working conditions on long-term change in work ability might be negligible. The small effects found in our 5-year follow-up study are in line with previous studies with shorter follow-up times, ranging from 2 weeks to 4 years (e.g., Martinez et al. 2016; McGonagle et al. 2015). Such small effect might be due to the relatively long causal pathway between working conditions and work ability, which could be mediated by factors such as early signs of musculoskeletal complaints and/or poor mental health (Ilmarinen et al. 2008; van den Berg et al. 2009). Only standing/walking, awkward body postures, carrying/lifting and control over working time were significantly associated with a decrease and an increase in work ability from baseline to follow-up, respectively, although the effects were small in size.

The present study suggests that a longer exposure to adverse working conditions is more strongly associated with a reduction in work ability. It also indicates that the impact of working conditions on work ability is substantially stronger in vulnerable subpopulations characterized by poor health. Specifically, job resources including possibilities for development, control over working time and quality of leadership, are more strongly associated with increased work ability in populations with poor health than in the general population.

### Methodological considerations

A methodical strength of the present study is that we employed a longitudinal design, which may alleviate some of the biases of cross-sectional studies (Taris and Kompier 2014; Zapf et al. 1996). Also, we examined a random sample of employees aged 31–60 years in employments subject to pay social contributions (i.e., except civil servants, self-employed workers and freelancers), which covered 80% of all employees in that age range (Rose et al. 2017).

Further, we used validated and established scales from the COPSOQ to cover a wide range of psychosocial working conditions. We examined the effect of psychosocial factors without combining them into higher-order factors. Merging indicators of demands or resources is reasonable if their effects have the same size and direction, but little is known as to whether this is the case (Burr and d’Errico 2018). From the point of view of preventive intervention, it is of special interest to gain knowledge about specific working conditions that should be improved to safeguard employees’ work ability.

This study has also some limitations worth considering. The response in the cohort sample was only 19%; however, it was independent of gender and only slightly smaller among younger age groups and in lower social classes. In addition, the response among those who took part in the follow-up interviews was only to a limited extent associated with baseline work ability. Thus, there are no indications of a strong bias due to attrition. Given the sampling procedure, we had no information about the association between working conditions and work ability in employees younger than 31 years or older than 60 years, as well as among those employees whose employers were not subject to mandatory social security contributions (this applies to civil servants, self-employed individuals and freelancers). The biases introduced by either the study sampling frame or non-response might have led to an underrepresentation of employees with poor working conditions, as these were correlated especially with low SEP (Table 4). However, we assume that such an underrepresentation would not affect the risk estimates, but solely lead to imprecise estimates in terms of wider confidence intervals.

Both predictors and outcomes were assessed by self-reports in a personal interview setting. This may have introduced common method variance, which is, however, reduced in longitudinal studies (Taris and Kompier 2014; Zapf et al. 1996).

The main analyses were limited to respondents who were still employed at follow-up, since unemployed respondents did not respond to all items of the WAI. Although this is a common approach in prospective studies focusing on work ability, it might introduce selection bias because the remaining sample is healthier (Schuring et al. 2019; van den Berg et al. 2010). As in any study on working populations, self-selection into occupations should also be considered.

We assessed duration of exposure using stability of employment as proxy. Both our study and recent research have shown that working conditions are more stable for those remaining in the same employment (Garthe and Hasselhorn 2020) (Appendix Table A). We refrained from directly estimating change by calculating changes in self-reported exposure to working conditions from baseline to follow-up, to minimize common method variance bias (Taris and Kompier 2014; Zapf et al. 1996). Studies with at least three measurement points could yield a better picture of duration of exposure than using employment trajectories as proxy. This would allow for using change in exposure at the first waves as predictors of change in work ability in the last waves.

We did not assess reverse causality, namely the effect of work ability on changes in job demands and resources; this mechanism could lead to an underestimation of effects in studies such as the present one. Cohorts with at least three measurement points could assess the effects of such selection processes (Beltagy et al. 2018; Taris and Kompier 2014).

We used a follow-up of 5 years, which may have resulted in an underestimation of the effects of working conditions on work ability. It has been shown that effects in follow-up studies tend to decline after 2 years (Ford et al. 2014).

### Comparison with other studies

The existing literature on antecedents of work ability is characterized by a wide methodological heterogeneity with respect to populations, measurement, analyses and reporting. For example, most of the linear regression-based studies did not report additional explained variances. Therefore, our main finding regarding the weak long-term effect of working conditions on work ability could only be confirmed by two studies reporting such additional explained variances, focusing on health among crowd and industrial workers in Brazil and the US (Carmen Martinez et al. 2016; McGonagle et al. 2015).

Focusing on the less investigated factors, in a Dutch study on construction workers lifting heavy loads was found in association with decreased work ability; however, no association was observed in a heterogeneous Norwegian worker sample and among Dutch industrial workers (Emberland and Knardahl 2015; Rongen et al. 2014; Tonnon et al. 2019). One Finnish study among food workers confirmed our results of an association between repetitive movements and decreased work ability (Oakman et al. 2019). Regarding work pace, for which we did not find associations with decreased work ability, we are not aware of previous studies focusing on this factor separately. As in our study, a US study on crowd workers also failed to find an association between amount of work and changes in work ability (McGonagle et al. 2015). We found an association between control over working time and increased work ability, but we are not aware of previous studies examining this factor separately. The association between role clarity and increased work ability we found in our study was also observed in the Norwegian study on a heterogeneous sample of workers mentioned above (Emberland and Knardahl 2015). Regarding the above mentioned less investigated factors, methodological variations prevent from making reliable comparisons between our findings and that of other studies.

Focusing on frequently investigated factors, such as awkward body postures, quantitative demands, influence at work, possibilities for development, social support, and quality of leadership, we generally did not find a high level of agreement between our study and previous studies (Table 1). Four studies found significant associations between awkward body postures and decreased work ability – the same was found in our study; however, the above-mentioned Norwegian study did not find such an association (Emberland and Knardahl 2015). In our study, we found no significant association between possibilities for development and increased work ability. However, such association was significant in all but one study; in the Norwegian study mentioned above, a significant U-shaped association was observed. We did not find a significant association between influence at work and work ability. The previous evidence regarding this association is mixed. Five studies, including the Norwegian one on a heterogeneous sample of workers (Emberland and Knardahl 2015), found significant associations; however, the remaining three, including the other Swedish study on a heterogeneous sample of workers (Leijon et al. 2017), failed to do so. We are not able to explain these deviating findings due to a number of methodological differences, including, for instance, mutual vs. no adjustment for other working conditions, heterogeneous vs. specific population, study size, linear vs. logistic regressions or use of the full WAI vs. subscales or single items.

### Duration of exposure

To our knowledge, only a few longitudinal studies examined the effect of duration of exposure to adverse working conditions on work ability (Garthe and Hasselhorn 2020; Tuomi et al. 2001, 1997, 2004). Most of these studies included exposure at follow-up (Tuomi et al. 2001, 1997, 2004), which could lead to an overestimation of the associations between the independent and the dependent variables due to common method variance (Taris and Kompier 2014). The issue of duration of exposure is crucial as most longitudinal studies – including the present one – rely on relatively long follow-up intervals. We tried to address this by conducting a sensitivity analysis limiting the sample to those employees who did not change their employment between baseline and follow-up. The associations we found were only slightly stronger than the associations observed in the main analysis. There is a need for more studies with improved assessments of duration of exposure to confirm the present the relatively small effects found in the present study.

### Health status

The present study suggests that working conditions have a stronger impact on work ability among employees with poor health. A previous study found a significant interaction between possibilities for development and health status in relation to work ability (Weber et al. 2020). Possibilities for development had a slightly stronger effect on subsequent work ability among employees with depressive symptoms than among those without. This result aligns with our finding that job resources such as possibilities for development play a stronger role in improving work ability among employees with poor than among employees with good health. A possible interaction between working conditions and health was previously considered also in studies on labour market participation and sickness absence, which by definition is related to work ability (Boot et al. 2014; de Boer et al. 2018; Jonsson et al. 2019). Both studies found stronger effects of working conditions among those with sickness absence than among those without.

Our findings suggest that there is merit in investigating factors that may increase individual vulnerability to working conditions, which, in turn, may lead to reduced work ability. Such factors may include baseline levels of self-rated health, sickness days, chronic disease, or work ability itself. The role of vulnerability is in line with recent studies indicating that psychosocial factors have a larger health impact among workers with early signs of impairment or disease (Holtermann et al. 2011; Kivimaki et al. 2018; Kivimaki and Steptoe 2018). Other factors such as age might modify the association between working conditions and work ability (Hellemans and Lapthorn 2016).

## Concluding remarks

We examined 5-year prospective associations between physical and psychosocial working conditions and changes in work ability in a sample of employees in Germany. We found that, in a random sample of employees, such long-term associations were weak, with only physical demands and control over working time being associated with small changes in work ability. Stronger prospective associations were found only in a subsample with a high number of self-reported sickness days; specifically, possibilities for development, control over working time and quality of leadership were associated with significant changes in work ability. We cannot rule out that stronger effects could have been obtained with shorter follow-up intervals or a better control of selection processes.

Further longitudinal studies conducted in various settings, including samples collected in other countries and occupational groups, are needed to confirm these findings.

Overall, four recommendations for future research can be drawn based on the results of the present study: (1) an increased focus on short-term effects; (2) the identification of vulnerable subgroups of employees; (3) the consideration of duration of exposure by, for instance, including repeated measurement points; (4) the inclusion of a broader range of individual physical and psychosocial working conditions.

From an intervention point of view, the results suggest that, to protect employees’ work ability, one should improve working conditions, especially by decreasing physical demands and increasing job resources such as control over working time. Second, employees with poor health should benefit from such interventions the most, especially when job resources are increased.

## Appendix

See Table A.

**Table A:** Associations between working conditions at baseline and follow-up stratified by employment stability during follow-up among 2,078 employees aged 31 to 60 years at baseline in Germany. Linear regressions

Domain	Working condition at baseline	Association to working condition at follow-up				
		Interaction with employment stability status, *p*^a^	Stayed in the same employment relationship during follow-up (*n* = 1,698)		Changed employment relationship during follow-up (*n* = 380)	
			Beta^b^	95% CI	Beta^b^	95% CI
Physical demands	Standing/walking	***0.000***	0.82	0.80–0.84	0.62	0.54–0.70
	Demanding body post	***0.003***	0.64		0.52	0.45–0.60
	Carrying/lifting	*0.770*	0.64	0.61–0.67 0.60–0.67	0.63	0.55–0.70
	Repetitive movements	***0.001***	0.44	0.40–0.49	0.27	0.18–0.36
Quantitative demands	Work pace	***0.028***	0.54	0.50–0.53	0.43	0.32–0.53
	Amount of work	***0.000***	0.59	0.56–0.63	0.40	0.30–0.49
Control	Influence at work	***0.000***	0.60	0.56–0.63	0.37	0.28–0.46
	Poss. for development	***0.000***	0.52	0.48–0.56	0.40	0.39–0.48
	Control over working time	***0.000***	0.73	0.70–0.76	0.52	0.44–0.61
Relations	Role clarity	*0.209*	0.38	0.34–0.42	0.31	0.22–0.41
	Quality of leadership	***0.000***	0.45	0.41–0.49	0.25	0.16–0.33

Significance level *p* = 0.05 (Rothman 1990)

^a^This *p* value denotes if the working condition at baseline and employment stability status during follow-up interacts as risk factors for the working condition at follow-up

^b^Denote observed associations between the working condition at baseline and the same working condition at follow-up
